# High-energy synchrotron-radiation-based X-ray micro-tomography enables non-destructive and micro-scale palaeohistological assessment of macro-scale fossil dinosaur bones

**DOI:** 10.1107/S1600577523001790

**Published:** 2023-04-07

**Authors:** Takuya Imai, Soki Hattori, Kentaro Uesugi, Masato Hoshino

**Affiliations:** a Institute of Dinosaur Research, Fukui Prefectural University, 4-1-1 Matsuoka Kenjojima, Eiheiji, Fukui 910-1195, Japan; b Japan Synchrotron Radiation Research Institute, 1-1-1 Kouto, Sayo, Hyogo 679-5198, Japan; Tohoku University, Japan

**Keywords:** dinosaurs, fossils, synchrotron radiation-based X-ray micro-tomography, virtual palaeohistology

## Abstract

Palaeohistological analysis has numerous applications in understanding the palaeobiology of extinct dinosaurs. Recent developments of synchrotron-radiation-based X-ray micro-tomography (SXMT) have allowed the non-destructive assessment of palaeohistological features in fossil skeletons. Yet, the application of the technique has been limited to specimens on the millimetre to micrometre scale because its high-resolution capacity has been obtained at the expense of a small field of view and low X-ray energy. Here, SXMT analyses of dinosaur bones with widths measuring ∼3 cm under a voxel size of ∼4 µm at beamline BL28B2 at SPring-8 (Hyogo, Japan) are reported, and the advantages of virtual-palaeohistological analyses with large field of view and high X-ray energy are explored. The analyses provide virtual thin-sections visualizing palaeohistological features comparable with those obtained by traditional palaeohistology. Namely, vascular canals, secondary osteons and lines of arrested growth are visible in the tomography images, while osteocyte lacunae are unobservable due to their micrometre-scale diameter. Virtual palaeohistology at BL28B2 is advantageous in being non-destructive, allowing multiple sampling within and across skeletal elements to exhaustively test the skeletal maturity of an animal. Continued SXMT experiments at SPring-8 should facilitate the development of SXMT experimental procedures and aid in understanding the paleobiology of extinct dinosaurs.

## Abstract 2

Synchrotron-radiation-based X-ray micro-tomography at beamline BL28B2 at SPring-8 (Hyogo, Japan) enables the non-destructive assessment of palaeohistological features in dense, fossilized bones of an allosauroid dinosaur, *Fukuiraptor kitadaniensis*, demonstrating its effectiveness in virtual palaeohistology.

## 1. Introduction

Histology, the study of tissues at the microscopic to sub-microscopic level (Bailleul *et al.*, 2019 ▸), is extensively applied to fossil dinosaur skeletons and has become essential in studying dinosaur remains to date, with its advantages demonstrated in numerous cases. For example, paleohistological techniques have allowed assessment of growth patterns and the physiology of various extinct dinosaurs, in particular providing essential information about the thermodynamics of non-avian theropods through their evolution toward birds (*e.g.* Padian *et al.*, 2001 ▸; Erickson, Rauhut *et al.*, 2009 ▸; Grady *et al.*, 2014 ▸). Additionally, palaeohistological analyses have enabled estimation of the age of individuals within a dinosaur assemblage, leading to inferences about the population structure and reconstruction of the population ecology for some dinosaur taxa (*e.g.* Erickson, Makovicky *et al.*, 2009 ▸; Woodward *et al.*, 2015 ▸; Bo *et al.*, 2016 ▸). Determining the age of individual dinosaurs through palaeohistological assessments has also helped to sort out dinosaur taxonomy when ontogenetic variation may complicate species identification (*e.g.* Horner & Goodwin, 2009 ▸; Fowler *et al.*, 2011 ▸; Hone *et al.*, 2016 ▸).

More recently, the development of synchrotron-radiation-based X-ray micro-tomography (SXMT) has allowed virtual acquisition and examination of palaeohistological sections. Palaeohistology through SXMT (hereinafter virtual palaeohistology to differentiate it from traditional palaeohistology) appears as effective as traditional palaeohistology in visualizing microscopic palaeohistological features including the lines of arrested growth, cementing lines, secondary osteons, Haversian canals, cell lacunae and medullary bone. For example, through a series of virtual-palaeohistological studies, ossification patterns, growth histories and geometries of muscle attachments are assessed in the humeri of fossil fish and early tetrapods (Sanchez *et al.*, 2012 ▸, 2013 ▸, 2014 ▸, 2016 ▸; Estefa *et al.*, 2020 ▸). Some additional studies also focus on Devonian fossil fish to understand the palaeobiology of the early vertebrates through virtual palaeohistology (Giles *et al.*, 2013 ▸; Qu *et al.*, 2015 ▸; Vaškaninová *et al.*, 2020 ▸; Bremer *et al.*, 2021 ▸). To date, virtual palaeohistology is extensively employed in studying various aspects of fossil hominids, including diet, dental development, life histoly and taxonomy (Smith *et al.*, 2007 ▸; Smith & Tafforeau, 2008 ▸; Tafforeau & Smith, 2008 ▸; Smith *et al.*, 2010 ▸; Tafforeau *et al.*, 2012 ▸; Le Cabec *et al.*, 2015 ▸; Smith *et al.*, 2015 ▸; Smith *et al.*, 2018 ▸; Beaudet *et al.*, 2021 ▸) (see Table S1 of the supporting information for a list of selected studies applying virtual palaeohistology to fossil vertebrate skeletons).

Virtual palaeohistology can be advantageous over traditional palaeohistology in that it causes no damage to samples and acquires multiple histological sections virtually for a sample of interest, although the cost of using SXMT and the proposal systems for applying for access to it can be challenging. Among numerous synchrotron facilities around the world, virtual-palaeohistological techniques have been demonstrated effective to fossil vertebrate remains at beamlines ID19 and BM05 at the European Synchrotron Radiation Facility (ESRF), at the Swiss Light Source and at beamline BL20B2 at SPring-8 (Table 1 ▸).

**Figure 1:**
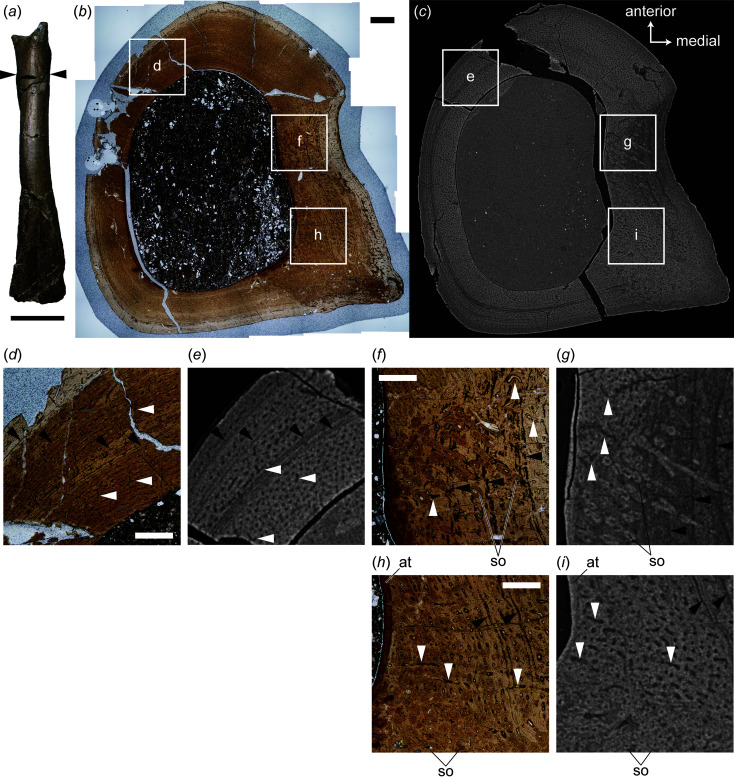
Exterior (*a*), transverse thin sections (*b*, *d*, *f*, *h*) and virtual-histological sections (*c*, *e*, *g*, *i*) of FPDM-V-10580 in anterior (*a*) and proximal (*b*)–(*i*) views. Arrowheads in (*a*) indicate the level at which each thin section was obtained. Rectangles in (*b*) and (*c*) indicate areas magnified in (*d*)–(*i*). Black arrowheads in (*d*)–(*i*) indicate lines of arrested growth. White arrowheads in (*d*)–(*g*) indicate reticular canals, whereas those in (*h*) and (*i*) indicate radial canals. Thin-section images and corresponding virtual-histological images are on the same scale. Abbreviations: at, avascular tissue; so, secondary osteon. Scale bars are equal to 30 mm for (*a*), 1 mm for (*b*), and 0.5 mm for (*d*), (*f*) and (*h*).

Despite the apparent usefulness and advantage of virtual palaeohistology on vertebrate fossils, its applications to non-avian dinosaur skeletal fossils are fairly limited to the following handful of cases. Dumont *et al.* (2016 ▸) applied SXMT to visualize histological microstructures of non-avian theropod teeth to compare them with those of avian teeth belonging to fossil toothed birds *Hesperornis* and *Ichthyornis*. Through SXMT analyses on dromaeosaurid *Halzkaraptor* to reveal its skeletal morphologies and neurovascular systems, Cau *et al.* (2017 ▸) notes that the virtual-histological sections capture a single line of arrested growth. Shen *et al.* (2019 ▸) has utilized SXMT at BL20B2 of SPring-8 to virtually analyse the forearm histology of troodontid *Daliansaurus* (measuring about 1 m in length head-to-tail). In addition to the above studies, although histological assessments are not involved, Voeten *et al.* (2018 ▸) present cortical canals in the limb bones of paravian *Archaeopteryx* and compsognathid *Compsognathus* through SXMT (see also Fig. 1 ▸, and Figs. S2 and S10 of the supporting information). While these studies demonstrate the effectiveness of virtual palaeohistology to non-avian dinosaurs, such attempts are far-limited compared with the cases in early vertebrates and fossil hominids.

This limitation may be due to the features of X-ray beams of synchrotron radiation facilities often being inadequate to analyse large-scale dinosaur bones. In general, X-ray tomography of centimetre-scale dense dinosaur bones requires a large field of view and a high-energy beam at the expense of resolution, resulting in a failure to capture target palaeohistological features in virtual-histological sections. Increasing the resolution leads to smaller fields of view on the millimetre-scale, which is too small to obtain virtual-histological sections equivalent in size to a traditional thin section [see Chapelle *et al.* (2020 ▸) for the ‘stitching’ technique of multiple tomographic slices from multiple scans into one large slice to compensate for the limited field of view of a single scan]. Another potential reason could simply be the limited awareness of virtual palaeohistology being effective to fossil vertebrate bones, as virtual palaeohistology is still a relatively new and developing technique. As reviewed above, there are a wealth of virtual-palaeohistological studies about early vertebrates and fossil hominids. In contrast to these examples, virtual palaeohistology is less frequently employed to analyse the palaeobiology of dinosaurs.

SPring-8 (Sayo, Hyogo, Japan) is one of the few synchrotron radiation facilities that enables virtual-palaeohistological analyses on dense, centimetre-scale dinosaur bones in reasonable time and computational labour (Table 1 ▸). Its high-energy X-ray beamline, BL28B2, equipped with a detector for large-field-of-view and micrometre-scale image resolution, and capable of generating a high-energy X-ray beam, is well suited for obtaining centimetre-scale virtual-histological sections of long dinosaur bones in a reasonable amount of time. In this study, we validate the effectiveness of BL28B2 for virtual palaeohistology by presenting palaeohistological features of a fossilized femur of a middle-sized theropod captured in virtual-histological sections. We compare the thin section images of the specimens obtained through traditional palaeohistological techniques with the virtual-histological sections and argue that virtual palaeohistology can reveal microscopic features of dinosaur bone matrix as effectively as the traditional techniques do. We further encourage application of virtual palaeohistology to various dinosaur fossils, considering its advantage in indestructively and exhaustively assessing the palaeohistology of valuable specimens to rigorously investigate their palaeobiology.

## 2. Materials and methods

### 2.1. Materials

Studied specimens include FPDM-V-10580 and -10581 (formerly 97080937 and 97081202, respectively; FPDM: Fukui Prefectural Dinosaur Museum) representing left and right femora, respectively, referred to as juvenile specimens of *Fukuiraptor kitadaniensis* (Azuma & Currie, 2000 ▸) based on a suite of morphological features characteristic of allosauroids (Currie & Azuma, 2006 ▸). FPDM-V-10580 and -10581 measure 10.9 cm in length and 2.9 cm in maximum width, and 19.6 cm in length and 4.6 cm in maximum width, respectively. The materials were yielded from the Lower Cretaceous (Aptian, 120 Ma) Kitadani Formation, Tetori Group, cropping out in the Kitadani Dinosaur Quarry, Katsuyama, Fukui, Japan. Each specimen was collected separately, and no evidence suggests they belong to the same individual of *F. kitadaniensis*. The bones exhibit minor signs of weathering in the form of longitudinal cracking [weathering stage 1 (*sensu* Behrensmeyer, 1978 ▸)].

### 2.2. Thin-section preparation

Transverse thin sections are sampled at the level of the fourth trochanter, an attachment site for the caudofemoral musculature, to observe a histological variation within the cortex [Figs. 1 ▸(*a*), 1(*h*)]. Computed tomography is applied to the bone near the cut section to obtain virtual-histological sections and compare them with traditional thin sections.

### 2.3. Computed tomography

High-energy X-ray microtomography at bending-magnet beamline BL28B2 at SPring-8 was used to observe dinosaur samples. BL28B2 is a sole general-purpose public beamline which utilizes white X-ray beam from the bending-magnet source without passing through any optical devices. To use the high-energy components in the white X-ray beam at BL28B2, lower-energy X-rays are removed by filtering with a heavy metal absorber, which is composed of a tungsten plate of thickness 500 µm and a lead plate of thickness 2 mm. Consequently, high-energy X-rays with a peak energy of ∼200 keV are applied for tomographic measurements (Hoshino *et al.*, 2017 ▸). The absorber was rotated in the plane perpendicular to the X-ray beam with a rotation speed of approximately 1800 r.p.m. to reduce the artefacts resulting from the uneven transmission of the absorber.

Each sample was placed in a plastic container, and the gap between the sample and the container was filled with melanin sponges for stability. The propagation distance from the sample to the X-ray imaging detector was set to 3 m. A visible-light conversion-type X-ray imaging detector composed of an AA60 beam monitor (Hamamatsu Photonics KK) was used to detect the X-ray transmission images. A Lu_3_Al_5_O_12_:Ce^+^ (LuAG) single crystal with a thickness of 500 µm was used as the scintillating material. A high-definition CMOS camera [C13949-50U, 4096 (H) × 3008 (V) pixels, 3.45 µm × 3.45 µm, 12-bit ADC, Hamamatsu Photonics KK] captured the visible-light image focused by the lens system. The magnification factor of the lens system was ×0.81. The effective pixel size of the projection image was 3.99 µm, which is slightly smaller than the calculated values. This is because the transmission image of the sample was slightly magnified by the long propagation distance. The effective field of view of the image was 16.3 mm (L) × 1.6 mm (V), where the vertical field of view is limited by the beam size of the high-energy X-rays.

An advantage of using X-rays from synchrotron radiation is the high degree of spatial coherence available even in the high-energy region. This is effective for X-ray imaging using refraction contrast, which is represented as an edge-enhancement effect in the image. For fossil samples, tomographic measurements with refraction contrast can be efficient to improve the density resolution.

In the tomographic measurements of our samples, both offset scan and normal scan methods were used. Under the normal scan method, the sample is rotated by 180° to obtain transmission images in SXMT. This is because the X-ray beam can be virtually treated as a parallel beam. In the offset scan method, the rotation axis is offset to the edge of the effective field of view in the projection image. In this method, only half of the sample is visible in each transmission image with respect to the rotation axis. Therefore, a 360° rotation is needed. In this case, pairs of images that are 180° apart are matching halves, which can be digitally combined to recreate whole images, resulting in a nearly doubled field of view compared with the normal scan method. In the present study, the amount of the offset of the rotation axis was set to 7.7 mm, resulting in the effective field of view being expanded to 31.7 mm (L). Each scan consisted of 3600 projections with an exposure time of 40 ms in both normal and offset scans. Note that this number of projections is lowered to reduce the acquisition time compared with what is considered adequate by Kak & Slaney (2001 ▸) and Withers *et al.* (2021 ▸). According to these studies, the recommended number of projections is equal to *q*π/2, where *q* is the number of pixels across the detector, giving around 6400 projections for our experimental condition. The total acquisition time for a scan was approximately 3 min in the normal scan and 6 min in the offset scan. Each sample was scanned three to five times in the vertical direction (parallel to the longitudinal direction of the long bone) step-by-step with a stepping amount of 1.42 mm.

### 2.4. Data processing

Tomographic reconstruction was carried out with homemade software based on a filtered back-projection method using a Chesler filter. Phase retrieval was not employed in this study. Tomography images for each sample were exported in 4096 × 4096 pixel (normal scan) and 7944 × 7944 pixel (offset scan), 16-bit tif format and filtered using Gaussian blur in *ImageJ* (filter size of σ*x* = σ*y* = σ*z* = 1.0) to reduce artificial noise. Then, a region of interest was cropped for further analyses. The images were not binned in order to keep the resolution. These images were then imported into *VGStudio 4.3* (Volume Graphics, Inc.) to modify image contrasts and generate the virtual thin-sections.

## 3. Results

### 3.1. Traditional thin-section palaeohistology

For FPDM-V-10580 [Fig. 1 ▸(*a*)], the transverse section at the level of the fourth trochanter exhibits a cortex mostly composed of the fibrolamellar bone [Fig. 1 ▸(*b*)]. Most vascular canals are arranged in the longitudinal orientation while some are in the reticular orientation [Figs. 1 ▸(*d*), 1(*f*)] in the medial aspect bearing the depression anterior to the fourth trochanter, in which some canals are oriented radially [Fig. 1 ▸(*h*)]. Internally, the anterior part of the medial aspect exhibits secondary osteons aligned toward the posteromedial direction [Fig. 1 ▸(*f*)]. The secondary osteons occupy the posteromedial corner bearing the fourth trochanter, and the area occupied by the secondary osteons becomes narrow in the external layer [Fig. 1 ▸(*b*)]. The endosteal surface is covered by a thin layer of avascular tissue except in the anterior side. There are four lines of arrested growth (LAGs) at most, while some parts of them are obscured by the secondary osteons.

**Figure 2:**
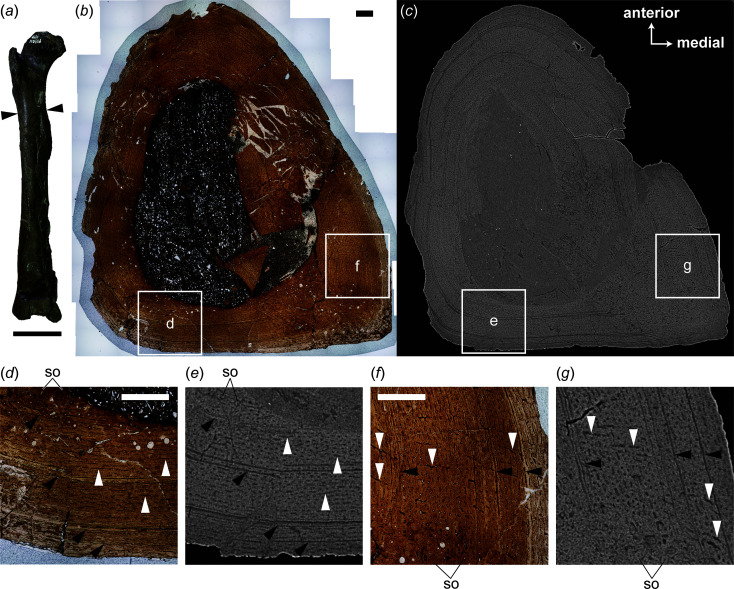
Exterior (*a*), transverse thin sections (*b*, *d*, *f*) and virtual-histological sections (*c*, *e*, *g*) of FPDM-V-10581 in anterior (*a*) and proximal (*b*)–(*g*) views. Arrowheads in (*a*) indicate the level at which each thin section was obtained. Rectangles in (*b*) and (*c*) indicate areas magnified in (*d*)–(*g*). Black arrowheads in (*d*)–(*g*) indicate lines of arrested growth. White arrowheads in (*d*) and (*e*) indicate laminar canals, whereas those in (*f*) and (*g*) indicate radial canals. Thin-section images and corresponding virtual-histological images are on the same scale. Abbreviations: so, secondary osteon. Scale bars are equal to 20 mm for (*a*) and 1 mm for (*b*), (*d*) and (*f*).

In the transverse section of FPDM-V-10581, the anteromedial part is fractured, and several minor fractures can be observed in the cortex throughout the circumference (Fig. 2 ▸). As in FPDM-V-10580, the cortex is mostly composed of the fibrolamellar bone. The vascular canals are mostly arranged in the longitudinal orientations and some are connected to each other to form laminar and radial canals [Figs. 2 ▸(*d*), 2(*f*)]. The secondary osteons are also seen in the inner part as a result of the resorption. Although the medial aspect of FPDM-V-10581 does not exhibit a fossa dorsal to the fourth trochanter superficially, the fragment in the medullary cavity exhibits the secondary osteons arranged in some lines directed posteromedially, like those seen in the medial aspect of FPDM-V-10580. While the deposition of avascular tissue on the endosteal surface is extremely thin, it is observable in the medial and posterolateral aspects. As in FPDM-V-10580, there are four LAGs at most, while some of them are obscured by the secondary osteons and the medullary cavity [Fig. 2 ▸(*d*)].

### 3.2. Virtual palaeohistology

Virtual-histological sections allow multiple histological features seen in the thin sections to be visualized. A deep, fibrolamellar cortex is visible as the layer encircling the darker medullary cavity, indicating that the former is denser than the minerals infilling the latter [Figs. 1 ▸(*c*), 2 ▸(*c*)]. Although the osteocyte lacunae are ambiguous in the virtual-histological sections, vascular canals of the primary osteons arranged in the longitudinal, reticular and radial patterns are apparent as micrometre-scale dark dots or lines in virtual-histological sections of the cortex, probably due to infilling diagenetic minerals less dense than the cortex compared with the primary bone matrix [Figs. 1 ▸(*e*), 1(*g*), 1(*i*), 2(*e*), 2(*g*)]. Therefore, our virtual-histological sections visualize arrangements of individual canals as well as their distribution within the cortex. Individual secondary osteons are observable and predominantly present in the posteromedial corner, as in the thin-section image [Figs. 1 ▸(*i*), 2 ▸(*g*)]. The thin, avascular endosteal deposit is distinguished by sparsely distributed vascular canals in the virtual-histological sections [Fig. 1 ▸(*i*)]. The LAGs corresponding to those seen in the thin-section images are also visible as black lines in virtual-histological sections.

## 4. Discussions

The present study employs SXMT on fossil bones of an allosauroid theropod, *Fukuiraptor kitadaniensis* (Currie & Azuma, 2006 ▸), at beamline BL28B2 at the synchrotron radiation facility SPring-8. The experiments demonstrate that SXMT conducted at BL28B2 allows virtual palaeohistology on centimetre-scale (about 12 cm in length and about 3 cm in width in this study) dinosaur fossils and renders multiple future applications. As a micro-scale analysis with a large field of view, the study is uncommon among virtual-palaeohistological studies conducted on vertebrate fossils (Table S1).

The virtual-histological sections generated by our SXMT experiments clearly capture certain osteohistological features in our samples including vascular canals, secondary osteons and lines of arrested growth. These features are identical to those observed using the traditional palaeohistological method with histological thin-sections. On the other hand, our SXMT analysis failed to visualize osteocyte lacunae and collagen fibre arrangements. This limitation likely comes from the effective pixel size (3.99 µm) used in the present experiment, which is too coarse for the lacunae typically of the order of a few micrometres in diameter (Williams *et al.*, 2021 ▸). The failure to capture the lacunae in the present analysis advocates for the importance of a multi-scale approach (micrometre to sub-micrometre scales) in conducting virtual histology under SXMT, which will be addressed in our future experiments.

As the synchrotron facility available for virtual-palaeohistological analyses of vertebrate skeletons, BL28B2 is advantageous in generating high-energy X-ray beam (∼200 keV) with micrometre-scale pixel size and large field of view. Combined with BL20B2 at SPring-8, which has demonstrated its effectiveness for virtual-palaeohistological analyses on vertebrate fossils at lower energy levels (∼100 keV) (Shen *et al.*, 2019 ▸; Monfroy *et al.*, 2022 ▸), BL28B2 should serve as a practical tool for investigating palaeohistological features of dense fossil vertebrate bones.

The non-destructive nature of virtual palaeohistology is sometimes advantageous relative to traditional palaeohistology. In traditional histological analyses, it is necessary to obtain a small piece of a fossil sample and grind it into a thin section (Padian & Lamm, 2013 ▸). This procedure unavoidably results in the loss of the sampled part, and can be problematic when applying to culturally or historically valuable specimens under conservation. On the other hand, it is important to conduct palaeohistological analyses on fossil dinosaur specimens to assess their maturity so that species identification and ecological inferences are properly assessed. Furthermore, in some cases, it may become necessary to address the maturity of previously described dinosaur specimens to revisit their taxonomic assignments and ecology (Horner & Goodwin, 2009 ▸; Fowler *et al.*, 2011 ▸; Hone *et al.*, 2016 ▸). Virtual palaeohistology through SXMT allows a practically non-destructive assessment of histological features when the aforementioned assessment becomes a necessity for valuable specimens whose destructive sampling cannot be permitted.

The non-destructive nature of virtual palaeohistology also enables multiple-data collection on specimens with minimum damage, allowing element-to-element and within-element palaeohistological comparisons. In the case of examining multiple histological sections, traditional palaeohistology requires sampling and processing of multiple bone tips. In virtual palaeohistology, multiple virtual-histological sections can be acquired simply by placing elements on a stage and conducting computed tomography analyses. This advantage is crucial in conclusively addressing the growth history of a dinosaur individual based on skeletal elements, as the number of observable LAGs and LAG-spacing can be variable within an element and an individual (Cullen *et al.*, 2014 ▸, 2020 ▸, 2021 ▸). For example, Cullen *et al.* (2014 ▸, 2020 ▸) finds that the number of LAGs and LAG spacing may vary across skeletal elements (*e.g.* femur versus phalanges) of an individual and recommends multiple sampling to conclusively address skeletal age based on these histological features. Also, Cullen *et al.* (2021 ▸) demonstrate that LAGs can split or merge within an element and argues for the importance of multi-sampling of different parts of the target element. Under SXMT, one can observe and compare LAGs of multiple virtual-histological sections of different elements and different parts of an element within an individual and obtain the maximum value, which corresponds to the maximum estimated skeletal age. This advantage can also facilitate assessment of the skeletal development of a dinosaur individual and understanding in what manner skeletal growth occurred for the individual, as well as the three-dimensional architecture of palaeohistological features in dinosaur bones.

Currently, the experimental settings for virtual palaeohistology vary from sample to sample and no experimental standards are available. This means that the experimental settings for any given analyses depend on previous experience and knowledge of the experimenters. To increase the effectiveness of virtual palaeohistology to various fossil skeletal elements, it is desired that future works in virtual palaeohistology share as much information about the experimental settings and the samples as possible. This practice would help future studies to set up experiments that best work for the specimens of interest. Fortunately, SPring-8 has two beamlines (BL20B2 and BL28B2) that are both adequate to conduct virtual palaeohistology on dinosaur bones (Table 1 ▸). It is hoped that continued SXMT experiments at the facility will aid in the development of experimental procedures for virtual palaeohistology on skeletal fossils and increase our understanding of the paleobiology of extinct dinosaurs.

## 5. Related literature

The following references, not cited in the main body of the paper, have been cited in the supporting information: Davesne *et al.* (2021 ▸); During *et al.* (2022 ▸); Gai (2018 ▸); Newham *et al.* (2020 ▸); Wang *et al.* (2019 ▸).

## Supplementary Material

Table S1: Selected studies on fossil vertebrate skeletons conducting virtual-palaeohistological analyses. DOI: 10.1107/S1600577523001790/mo5265sup1.pdf

## Figures and Tables

**Table 1:** Summarized list of synchrotron facilities and beamlines that have been utilized for virtual palaeohistology of fossil vertebrate bones

Facility	Beamline	Voxel size possible (µm)	Pixel size of detector	Energy range (keV)
European Synchrotron Radiation Facility, France	ID19	0.4–40	2048 × 2048	10–250
			2560 × 2160	
	BM05	1.4–15	2048 × 2048	6–60
			2560 × 2160	
Swiss Light Source, Switzerland	Tomcat	0.16–6.5	2560 × 2160	8–45
Diamond Light Source, UK	I12	1.3–18.53	2560 × 2160	53–150
	I13-2	0.325–2.6	2560 × 2160	8–30
SPring-8, Japan	BL20B2	1.0–12.0	2048 × 2048	13.5–113.3
	BL28B2	2.0–12.0	4096 × 3008	5–200
