# Supplementary material for: High-energy synchrotron-radiation-based X-ray micro-tomography enables non-destructive and micro-scale palaeohistological assessment of macro-scale fossil dinosaur bones
Source: J Synchrotron Radiat. 2023 Apr 7;30(Pt 3):627–33. doi: 10.1107/S1600577523001790 (PMC10161879; doi:10.1107/S1600577523001790)
Supplement: Supplementary file 1 [file s-30-00627-sup1.pdf]

JOURNAL OF SYNCHROTRON RADIATION

Volume 30 (2023)

Supporting information for article:

High-energy synchrotron-radiation-based X-ray micro-tomography enables non-destructive and micro-scale palaeohistological assessment of macro-scale fossil dinosaur bones

Takuya Imai, Soki Hattori, Kentaro Uesugi and Masato Hoshino

Table S1 Selected studies on fossil vertebrate skeletons conducting virtual-palaeohistological analyses.

Group	Taxon	Facility	Beamline	Material	Voxel (µm)	Detector Pixel Size	FOV (L) (mm)	Energy (keV)	Range (degrees)	Exposure (sec)	Projections (times)	Propagation (mm)	References
Vertebrates	*Shuyu zhejiangensis* (agnathan)	SLS	Tomcat	Skull	3.5	2048*2048	7.00	8–45	180	0.1	1501	?	Gai, 2018
	Thyestiida (osteostracans)	ESRF	ID19	Dermal skeleton	0.761	2048*2048	1.56	69	360	0.1	2999	100	Bremer et al., 2021
	Osteostraci	ESRF	ID19	Scale	0.28–0.56	2048*2048	0.57–1.15	19	?	0.3–0.4	1500	?	Qu et al., 2015
	Compagopiscis croucheri (placoderm)	ESRF	ID19	Humerus	0.678	2048*2048	1.39–2.29	30–123	180–360	0.4–2.0	1500–6000	10–950	Sanchez et al., 2012: Sanchez et al., 2013
	Placodermi	SLS	Tomcat	Dermal skeleton	0.74	2048*2048	1.52	21.5	180	?	1501	?	Giles et al., 2013
	Radotina tesselata and *Kosoraspis peckai* (placoderms)	ESRF	ID19	Tooth/skull	0.72	2560*2160	1.84	~124	360	0.25	6000	300	Vaškaninová et al., 2020
	Actinopterygii	ESRF<br>DLS	ID19<br>I13-2	Dentary	0.716	2560*2160	1.83	19–112	360	0.03–0.2	2999	20–220	Davesne et al., 2021
	Acipenseriformes (actinopterygian)	ESRF	BM05	Dentary/pectoral-fin spines	4.35	2048*2048	8.91	92	360	0.06	4998	1.5	During et al., 2022
	Eusthenopteron foordi (sarcopterygian)	ESRF	ID19	Humerus	0.678	2048*2048	1.39–2.29	30–123	360	0.4–2.0	1500–6000	5–950	Sanchez et al., 2012: Sanchez et al., 2013; Sanchez et al., 2014
Tetrapodomorphs	*Acanthostega gunnari*	ESRF	ID19	Humerus	0.678–1.12	2048*2048	1.39–2.29	30–123	360	0.4–2.0	1500–6000	5–950	Sanchez et al., 2016
	Seymouria sanjuanensis and *Discosauriscus austriacus* (seymouriamorphs)	ESRF	BM05	Humerus	3.03	2560*2160	7.76	120	360	0.1	6000	1360	Estefa et al., 2020
Dinosaurs	*Richardoestesia isosceles* (coelurosaur)	ESRF	ID19	Tooth	1.28	2048*2048	2.62	31–53	180–360	0.15–0.3	2499–5000	200	Dumont et al., 2016
	Compsognathus longipes (coelurosaur) and aff. *Deinonychus antirrhopus* (maniraptor)	ESRF	BM05	Humerus/ulna	6.43–6.46	2048*2048–2560*2160	1.32–1.65	88–160	360	0.04–0.25	6000	225–2100	Voeten et al., 2018
	Daliansaurus liaoningensis (maniraptor)	SPring-8	BL20B2	Ulna/radius	2.75	2048*2048	11.26	30	180	0.12	1800	300	Shen et al., 2019
	Halzkaraptor escuilliei (maniraptor)	ESRF	BM05	Tibia/radius	2.25	2560*2160	5.76	100	360	0.15	6000	700	Cau et al., 2017
	**cf. *Fukuiraptor kitadaniensis* (megaraptoran)**	**SPring-8**	**BL28B2**	**Femur**	**3.99**	**4096*3008**	**16.34**	**200**	**180–360**	**0.04**	**3600**	**3000**	**This Study**

		Avialans	*Archaeopteryx lithographica*	ESRF	ID19	Humerus/ ulna	3.11–6.56	2048*2048–2560*2160	6.37–16.79	129–184	360	0.15–0.32	4998–6000	160–900	Voeten et al., 2018
			Yanornis martini (enantiornithine)	SPring-8	BL20B2	Tibia/pubis	2.75	2048*2048	11.26	37.7	180	0.12	1800	300	Wang et al., 2019; Monfroy et al., 2022
			Hesperornis regalis and *Ichthyornis disper* (ornithurines)	ESRF	ID19	Tooth/dentary	1.28–3.50	2048*2048	2.62–7.16	31–53	180–360	0.15–0.3	2499–5000	200	Dumont et al., 2016
	Mammaliaforms		*Morganucodon watosoni* and *Kuehneotherium* spp.	ESRF SLS	ID19 Tomcat	Tooth/dentary	0.28–0.70	2560*2160	0.71–1.79	20–26.5	360	0.15–0.300	1500–2499	14–405	Newham et al., 2021
		Hominids	*Australopithecus prometheus*	DLS	I12	Tooth	3.25–7.91	2560*2160	8.32–20.25	140	360	0.25	9000	?	Beaudet et al., 2021
			Hominidae	ESRF	ID19	Tooth	0.60–4.96	2048*2048	1.22–10.15	30–167	180–360	0.3–0.9	150–5000	200–4000	Smith et al., 2007; Smith & Tafforeau, 2008; Tafforeau & Smith, 2008; Smith et al., 2010; Tafforeau et al., 2012; Le Cabec et al., 2015; Smith et al., 2015; Smith et al., 2018
